# An Observational Study Protocol for Assessing Lactation Intensity and Reduction in the Prevalence of Metabolic Syndrome After a Maternal Complication of Pregnancy (LEMON Study)

**DOI:** 10.1177/08903344251396545

**Published:** 2026-02-08

**Authors:** Maleesa M. Pathirana, Prabha H. Andraweera, Emily Aldridge, Melanie R. Wittwer, Susan Sierp, Gustaaf Dekker, Margaret A. Arstall

**Affiliations:** 1 Robinson Research Institute, University of Adelaide, Adelaide SA, Australia; 2 Department of Cardiology, Lyell McEwin Hospital, Adelaide, SA, Australia; 3 Department of Women’s Health, Lyell McEwin Hospital, Adelaide, SA, Australia

**Keywords:** breastfeeding, lactation, metabolic syndrome, observational study, pregnancy complications

## Abstract

**Background:**

Major pregnancy complications, such as hypertensive disorders of pregnancy, gestational diabetes mellitus, delivery of a small for gestational age infant, preterm delivery, and placental abruption, are associated with the development of Type II diabetes mellitus and cardiovascular disease. Improving breastfeeding longevity may reduce long-term risk for cardiovascular disease for those with major pregnancy complications. Metabolic syndrome (i.e., the presence of at least three specific cardiovascular disease risk factors) is an appropriate marker to assess future cardiovascular risk in the early postpartum.

**Research Aim:**

To determine whether lactation intensity in the first 6 months postpartum is associated with metabolic syndrome at 6 months postpartum among people who previously had a major pregnancy complication.

**Planned Analysis:**

This is a prospective observational cohort study is being conducted at a tertiary hospital in Adelaide, South Australia (ANZCTN12624001351505). We will be recruiting a total of 250 participants referred to a postpartum cardiovascular clinic after experiencing a major complication in pregnancy. To assess lactation intensity, participants complete online questionnaires on infant feeding patterns for 6 months postpartum. We will examine the relationship between lactation intensity and the development of metabolic syndrome at 6 months postpartum via binary logistic regression.

**Discussion:**

Results of this study aid our understanding of the complex relationship between major pregnancy complications, early postpartum metabolic syndrome, and lactation. This data will inform whether interventions to support lactation should be leveraged to prevent primary cardiovascular disease in these high-risk pregnancy cohorts.

## Background

Cardiovascular disease (CVD) is a leading cause of death among Australians assigned female at birth, with hospitalization rates for females with CVD continuing to increase (Australian Institute of Health and Welfare, 2023). There has been a significant increase in hospitalization for stroke in women younger than 55 years of age compared to men (Kelly et al., 2024). As premature CVD (< 45 years of age) is becoming more prevalent in people of reproductive age assigned female at birth, it is important to identify those who may be at high risk for its development. However, this is currently difficult within clinical practice, as conventional cardiovascular risk screening, such as the Framingham risk score (D’Agostino et al., 2008), underrepresents risk for those under 30 and is structured based on male-specific risk factors. Greater consideration is needed to identify a different pathway of prevention and cardiac care for those of reproductive age assigned female at birth.

Major pregnancy complications, such as hypertensive disorders of pregnancy (HDP), gestational diabetes mellitus (GDM), delivery of a small for gestational age infant, delivery of a preterm infant, and placental abruption, are all independently associated with a two-fold increased risk of coronary heart disease later in life (Parikh et al., 2021). Preeclampsia is also associated with a higher increased risk of stroke and cardiovascular mortality within 10 years postpartum (R. Wu et al., 2020). Clinical guidelines now recommend primary management of CVD risk for those assigned female at birth with the above pregnancy complications, by screening for CVD risk factors in early postpartum and supporting lifestyle changes, such as encouraging diet modification and exercise (Garovic et al., 2022; Lewey et al., 2024). However, studies assessing postpartum lifestyle interventions in people with HDP and GDM have shown that the interventions have varied efficacy, so these alone may not lead to a significant reduction in CVD risk (Hauspurg et al., 2023; Ukke et al., 2024). A qualitative study in Australia found that women with previous GDM find it difficult to engage in lifestyle interventions due to work commitments, available time, and the cost and difficulty of organizing child care (Sabag et al., 2023). Therefore, identifying additional strategies that can form part of a parental routine in the early postpartum period is necessary to reduce or delay future CVD.

Breastfeeding is mutually beneficial for both breastfeeding parent and child, with the World Health Organization (WHO) recommending breastfeeding exclusively for 6 months (WHO, 2011). It has been shown that lactating for over 12 months promotes a significant reduction in both chronic hypertension and diabetes (Rameez et al., 2019). This evidence forms the basis of the “reset hypothesis,” whereby the metabolic maladaptation of pregnancy, in the case of those with a major pregnancy complication, reverses more quickly and completely with lactation (Stuebe & Rich-Edwards, 2009). There is evidence to suggest that lactation improves CVD outcomes in individuals with previous HDP and GDM (Magnus et al., 2023; Pathirana, Ali, et al., 2021). However, there are still methodological discrepancies between studies on how breastfeeding or lactation status is reported and ascertained (Gunderson et al., 2015). While the WHO defines exclusive breastfeeding as feeding breastmilk with no other liquids or solids, this is not necessarily reflected in the literature. For example, Magnus et al. (2023) defined the duration of exclusive breastfeeding as the number of months of reported breastfeeding until the participant introduced formula or other milk, which does not include if alternate food or liquid (not other milk or formula) is introduced in addition to breastmilk. In this case, it is not known whether any breastfeeding still occurred in complement to alternative milk and introducing complementary foods. Therefore, the breastfeeding intensity across the course of a parent’s breastfeeding journey cannot be calculated. Even a partial level of breastfeeding may be cardioprotective; however, this has not been fully investigated. Furthermore, the variation of lactation definitions and the collection of data in a retrospective manner in the literature leads to inconsistent evidence, which is difficult to translate clinically (Mazhar et al., 2025).

Australian data has shown that only 37% of individuals who breastfeed are exclusively breastfeeding at 3 months postpartum (Australian Bureau of Statistics, 2022). Additionally, the rate of exclusive breastfeeding is likely to be lower for women with pregnancy complications (Horsley et al., 2022). Previous research has found that having GDM reduces breastfeeding self-efficacy, potentially because impaired glucose tolerance and suboptimal insulin levels impair the lactation pathway (Doughty et al., 2023). Therefore, barriers to adequate lactation, including cardiometabolic risk factors, lifestyle, and psychosocial factors during pregnancy need to be assessed.

Metabolic syndrome (MetS; diagnosed in the presence of obesity and two or more of the following: hypertension, Type II diabetes mellitus [T2DM], elevated triglycerides or reduced HDL-C) is a cluster of the most dangerous coronary heart disease risk factors, and is a precursor of T2DM and coronary artery disease (Alberti et al., 2009). People of reproductive age who were assigned female at birth have been shown to exhibit risk factors of metabolic syndrome, which can be used as a proxy marker to predict risk of premature CAD. Furthermore, people with a history of GDM have a 2-fold increased risk of having metabolic syndrome as early as 1 year postpartum (Pathirana, Lassi, et al., 2021). A systematic review and meta-analysis by Tørris & Bjørnnes (2020) revealed that lactation duration was associated with improvement of metabolic health and may play a protective role against MetS (Tørris & Bjørnnes, 2020). Lactation may confer benefits for multiple cardiovascular risk factors and MetS may be a useful outcome to measure improvement in cardiovascular health and assess the effect of lactation on all cardiovascular risk factors in people with a previous complication of pregnancy.

The aim of this study is to determine whether lactation intensity during the first 6 months is associated with the incidence of metabolic syndrome at 6 months postpartum among those with a previous major pregnancy complication. We hypothesize that individuals with major complications of pregnancy and a high lactation intensity (i.e., feeding their infant human milk for ≥ 80% of their feeds) in the first 6 months postpartum will be less likely to develop metabolic syndrome by 6 months postpartum.

### Primary Objective

1. To assess the relationship between lactation intensity and postpartum metabolic syndrome in women with major complications of pregnancy.

### Secondary Objectives

1. To determine the relationship between lactation intensity in the first 6 months postpartum and components of metabolic syndrome (i.e., peripheral blood pressure, dyslipidemia, glycemia, and waist circumference) in the same cohort.
2. To determine if lactation intensity affects other markers of cardiovascular risk (i.e., body mass index, serum insulin, and elevated high-sensitivity C-reactive protein) at 6 months postpartum in individuals with a previous complication of pregnancy.
3. To assess whether lactation intensity in the first 6 months postpartum affects non-conventional markers of vascular health such as augmentation index, central blood pressure, and mean arterial pressure in the same cohort.

### Exploratory Objective

1. To assess whether cardiometabolic, psychosocial, and labor and delivery variables during pregnancy affect lactation intensity in individuals with a previous complication of pregnancy.

## Methods

### Research Design

**Figure 1.**
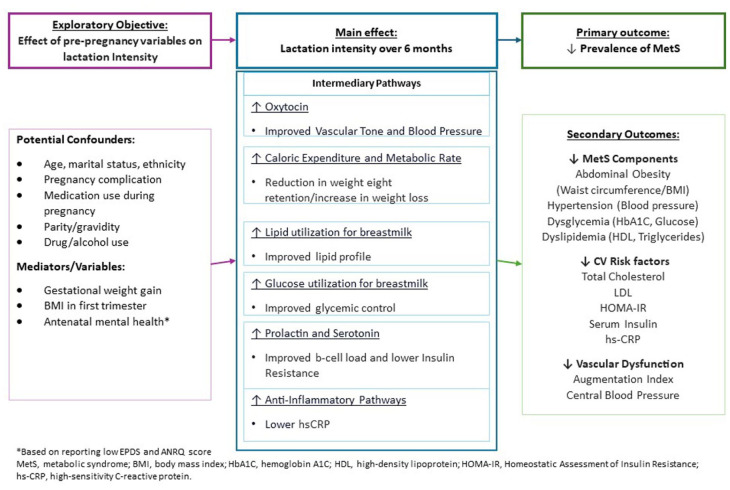
Theoretical Model_Clean.

This a prospective, observational cohort study based at a tertiary hospital in Adelaide, South Australia. The theoretical framework for this study is highlighted in Figure 1. In brief, the rationale for our study design is to assess the effect of lactation intensity over 6 months and the prevalence of metabolic syndrome at 6 months postpartum amongst participants with previous complications of pregnancy. This design also allows us to determine pregnancy and postpartum covariates that influence this association, focusing on variables that have a bidirectional association with lactation. Additionally, we will be able to understand the trend in lactation intensity over 6 months for those who experience more than one pregnancy complication, which has not yet been explored. This study is being conducted as per the National Health and Medical Research Council (NHMRC) National Statement on Ethical Conduct in Human Research. The study has been approved by the local Human Research Ethics Committee (HREC/18824). The study is registered with the Australian New Zealand Clinical Trials Network (ANZCTN12624001351505).

### Setting and Relevant Content

Pregnant patients being enrolled in this study undergo their antenatal care at a public tertiary acute-care hospital in Adelaide South Australia. This hospital services 400,000 patients residing in the Northern Adelaide Local Health Network (NALHN) region, which has some of the highest rates of coronary artery disease, diabetes, smoking and obesity in metropolitan Australia (greater than more populated states like Victoria and New South Wales (Social Health Atlas, 2021). This region is characterized as socioeconomically disadvantaged, which is reflected in poor health outcomes. In context, based on the Australian Bureau of Statistics Socioeconomic Index for Areas (SEIFA) index of relative socioeconomic disadvantage (SEIFA-IRSD) score (regions are scored as Quintiles 1–5, where Quintile 1 is the most disadvantaged), this hospital is located in the Playford government area, which is classified as Quintile 1 (Australian Bureau of Statistics, 2023).

This tertiary hospital provides obstetric care, adult cardiac and intensive services, and neonatal special care for infants born at ≥ 32 weeks gestation. The obstetrics department at the hospital has approximately 4,000 births annually. More than 50% of patients who deliver at this hospital experience a major complication of pregnancy, with gestational diabetes mellitus being the most prevalent. This is higher than the national average of 25%–30%. Women who have had a pregnancy complication are referred by the obstetrics team post-delivery to a postpartum cardiovascular preventative clinic, led by a nurse-practitioner, addressing postpartum healthy lifestyle interventions. Referral indications for the clinic include preeclampsia, gestational hypertension, GDM, preterm delivery, delivery of a small for gestational age infant, and placental abruption. Patients are recruited to this study by the study team once referral from the obstetrics team has been received and accepted by the clinic’s nurse practitioner.

Characteristics of the patients who attended the postpartum cardiovascular clinic from 2018 to 2021 have been reported previously (Aldridge et al., 2022). The average age of this cohort is 32.8 years, with 54.7% of the cohort being Caucasian, 20.2% of East Asian descent, 8.9% Middle Eastern, 8% Indian Subcontinent, 5.7% African, 4% Aboriginal and 0.8% Hispanic. In our cohort, 90.2% reported being either married or in a de-facto relationship. Only 25% of participants have a Bachelors’ degree or graduate degree, 38% have a trade certificate/diploma (equivalent to community college), and 20% have a high school education or lower. Approximately 6.5% of the patients in the clinic within this 4-year time period had hypertension at 6 months postpartum, and 5% had T2DM at the same time point. The rate of current smoking in clinic patients is 9.8%, with a previous smoking history for 18% (not published). In all, 40% of this cohort were reported to have MetS.

Breastfeeding initiation rates in Australia are high, with 96% of infants being breastfed at least once. At 6 months of age, 74% of infants are still receiving breastmilk (either exclusively or mixed (Australian Bureau of Statistics, 2022). In South Australia, the rate of exclusive breastfeeding at 6 months is similar to the national average of 33%–35%, and the rate of any breastfeeding (both mixed and exclusive) at 6 months is 71%. Individuals who are obese (i.e., BMI > 30 kg/m^2^), those who deliver preterm or small-for-gestational age infants, and those of lower socioeconomic status (such as those who deliver at our hospital) are significantly less likely to initiate and continue breastfeeding or exclusively breastfeed for 6 months postpartum (Australian Bureau of Statistics, 2022). Approximately 89% of the cohort in the postpartum cardiovascular preventative clinic initiate breastfeeding, with 52% of participants still breastfeeding at 6 months postpartum (either mixed or exclusively; not published). This is lower than Australia’s national rates of breastfeeding at these timepoints.

### Sample

#### Inclusion and exclusion criteria

Individuals are eligible to participate in this ongoing study if they are aged 18–45 years; are English-speaking in order to provide informed consent; have delivered at the study site, which is a tertiary hospital (regardless of parity); and have been referred to the postpartum healthy lifestyle clinic by the treating obstetric team for one or more of the following complications of pregnancy:

- Hypertensive disorders of pregnancy (including gestational hypertension, preeclampsia, eclampsia, and HELLP syndrome), requiring medical therapy or resulting in delivery at < 37 weeks’ gestation;
- Gestational diabetes mellitus requiring metformin or insulin therapy;
- Preterm delivery at < 35 weeks’ gestation;
- Delivery of a small for gestational age infant (< 5^th^ centile);
- Placental abruption.

Individuals are excluded if they cannot provide consent due to cognitive difficulty or intellectual disability, and if they have experienced fetal demise.

Referred patients are contacted by the research team about the study by phone 2 to 4 weeks after they have been discharged from their delivery hospitalization, and once the patient’s referral has been triaged by the clinic’s nurse practitioner. This timeframe allows patients to recover from their birth without being disturbed by the study team. The study team consists of primary and co-investigators who are clinicians and medical scientists working in the postpartum clinic. Potential participants are asked if they would like to participate in an online study where they complete monthly questionnaires on infant feeding frequency and current medical history. Potential participants are told that participating in the study is optional and does not affect their attendance at the postpartum healthy lifestyle clinic, as clinic attendance is part of their routine care.

#### Sample size

Based on a cohort of Australian women with previous pregnancy complications, with 80% power, a sample size of 250 women is required to detect an absolute reduction of 16.9% (from 38.1% to 21.2%) in the proportion of women who have metabolic syndrome who breastfed to at least 6 months postpartum compared to those who did not breastfeed to at least 6 months in the study cohort. To allow for a 15% loss to follow-up of participants, 280 women will be recruited (Aldridge et al., 2022).

### Data Collection

This study was approved by the local ethics committee and began recruiting participants in August 2023. We are still recruiting as of May 29, 2025. This study is anticipated to be completed by January 2026. Those who agree to participate undergo informed consent and sign an electronic Participant Information Sheet and Consent Form (PISCF) on Qualtrics.

Those who consent to participate in this study are asked to complete an infant feeding frequency questionnaire once a month for 6 months. Participant contact points and assessments are summarized in Table 1. Participants are sent the Infant Feeding Intentions scale (adapted from Nommsen-Rivers and Dewey, 2009; see Appendix 1 in the online supplemental material) to understand how they plan to feed their baby. This is done once at the start of the study period. Participants can choose to either receive an email or SMS survey link to a Qualtrics questionnaire, which includes one section about self-reported general health and medication use, current issues with breastfeeding, and whether a participant is receiving support for breastfeeding. The second section asks questions on the frequency of feeding human milk (i.e., by breastfeeding and/or expressing), formula, and/or mixed feeds (see Supplemental Material 1). Section 2 of the survey is based on the methods of Piper and Parks (2001) for ascertaining lactation intensity (Piper & Parks, 2001). Definitions of infant feeding methods are described in Table 2. To ensure that the questionnaires were readable, appropriate for breastfeeding parents, and understandable, they were reviewed by a consumer who was breastfeeding at the time they reviewed the documents.

**Table 1.** Participant Assessments and Study Timeline.

	Study Timeline						
Event	Discharge	1 Month Postpartum	2 Months Postpartum	3 Months Postpartum	4 Months Postpartum	5 Months Postpartum	6 Months Postpartum
Referral to Postpartum Clinic	×						
Informed Consent (Via Qualtrics)	×						
Infant Feeding Intentions Questionnaire (Via Qualtrics)		×					
Monthly Infant Feeding Questionnaire (Via Qualtrics)		×	×	×	×	×	×
Outpatient Appointment and Cardiovascular Screening (In-Person Attendance)							×

**Table 2.** Definitions of Infant Feeding Types for the Questionnaire.

Term	Definition
Breastfeeding	Breastfeeding is defined as lactation, which includes both breastfeeding and expressing human milk through pumping, then feeding this to your own baby.
Formula Feeding	Feeding your infant formula.
Solid	Feeding your infant solid foods.

At 6 months postpartum, participants attend the outpatient healthy lifestyle clinic appointment as part of their routine care. In brief, participants attend a nurse-practitioner-led, medical-scientist-supported outpatient clinic. The medical scientist collects information on medical and obstetric history, including details of the index pregnancy and past pregnancies, if relevant, to include in the clinical registry. Participants are asked for updated demographic information (age, marital status, employment, combined household income, post code, and socioeconomic status), medical history, medication use, obstetric and gynecological history, alcohol and drug use, diet, and exercise. The nurse practitioner then conducts a full cardiovascular screening assessment for cardiometabolic risk factors. With consent, participants’ medical and obstetric outcome data are abstracted from the clinic registry for the research study.

#### Data management

All data is kept confidential, and participants are informed of this as part of the informed consent process. Only the study research team has access to all electronic and paper data. Personal demographic information, questionnaire data, and clinical data is de-identified and replaced with a study code prior to analysis.

Participants are assigned a study number upon consent, and this is linked to their Qualtrics questionnaires. Qualtrics is an encrypted, web-based application that is password-protected for reliable and secure data storage. Only approved investigators can access the database. Clinic information is collected from the abstraction of physical and electronic medical records and in-person questionnaires, as mentioned above. Clinic data is linked to the study data, and all information is de-identified prior to analysis.

### Outcomes and Measurement

We have described our Theoretical Model of this study in Figure 1.

*Ascertainment of lactation intensity:* To determine lactation intensity, participants are asked about the frequency of different feeding types as described in Table 2.

Frequency of lactation is ascertained using a calculated intensity ratio devised by Piper and Parks, which assesses the number of breastfeeds (either breastfeeding and/or expressed breastmilk fed through a bottle) per day in relation to all other forms of feeding per day. Monthly scores are calculated between 0 (no lactation) to 1 (exclusive lactation) based on an average 24 hours of infant feeding in the 7 days from when the questionnaire was sent (i.e., the date on which the baby was born). At 6 months, the overall lactation intensity score is calculated as the average of the six intensity scores from 1 to 6 months (Piper & Parks, 2001). Lactation intensity is stratified across categories, with high lactation intensity being a score of 0.80 (i.e., representing ≥ 80% of all infant feeds across 6 months being breastmilk), median lactation intensity scoring 0.40–0.79 (i.e., representing 40%–79% of all infant feeds being breastmilk) and a low lactation being an intensity score of < 0.39 (i.e., < 39% of infant feeds over 6 months being breastmilk). We will report continuous lactation intensity scores and the lactation intensity categories. To conduct binary regression analyses to assess the association between high lactation intensity and prevalence of metabolic syndrome at 6 months postpartum, we will also report a dichotomized ratio, where scores are assessed as high intensity (≥ 0.80, i.e., ≥ 80%) versus non-high intensity (i.e., both low and median lactation intensity; ≤ 0.79, i.e., ≤ 79%). To assess the 6-month mean intensity score, we will look at the rate of participants who exclusively breastfed for 6 months (Lactation Score of 1) and those who never breastfed (Score of 0).

The primary outcome is the prevalence of MetS at 6 months postpartum. MetS diagnosis is based on a participant having any three of five of the Harmonizing the Metabolic Syndrome Criteria (Alberti et al., 2009) (Table 3), as measured by a trained nurse practitioner at the 6-month study visit:

**Table 3.** Definition of Metabolic Syndrome (Alberti et al., 2009).

Metabolic Syndrome (Harmonizing Definition) Requires Any Three of the Five Following:
1. Marker of adiposity or waist circumference ≥ 80cm (ethnicity-specific waist circumference values are the same across all ethnicities)
2. Raised triglycerides: ≥ 1.7 mmol/L or specific treatment for this lipid abnormality
3. Reduced HDL cholesterol: < 1.29 mmol/L or treatment for this lipid abnormality
4. Raised blood pressure: systolic BP ≥ 130 mmHg or diastolic BP ≥ 85 mmHg, or treatment of previously diagnosed hypertension
5. Raised fasting plasma glucose: ≥ 5.6 mmol/L or previously diagnosed T2DM.

After the participant has been seated for 20 minutes, their peripheral blood pressure is checked with the USCOM BP+ (Sydney, Australia). Height, weight, waist circumference (to the nearest 0.1 cm) are recorded once by the clinic’s medical scientist. Participants complete a fasting blood test and urinalysis prior to attending their appointment for cardiovascular risk screening. This blood test includes full blood count, high sensitivity C-reactive protein (hsCRP), electrolytes, renal function, liver function, lipids (total cholesterol, high-density lipoprotein [HDL], and non-HDL lipids), fasting glucose, glycosylated hemoglobin (HbA1c) and serum insulin. A urine specimen is collected to assess albumin, creatinine, and urine microscopy. These values will be used to assess measurements against the cut-offs for components of metabolic syndrome as part of our primary objective.

To assess our first secondary objective of determining the relationship between lactation intensity and components of metabolic syndrome, we will report the individual components of metabolic syndrome as above (i.e., abdominal obesity, peripheral blood pressure serum triglycerides, HDL, HbA1c, and serum glucose). For our next secondary objective, we will assess non-conventional vascular health markers, measured using the USCOM BP+, which non-invasively measures central blood pressure and augmentation index (i.e., a marker of arterial stiffness).

Other secondary outcomes include measuring components of cardiovascular health that increase the CVD risk, such as total cholesterol, low density lipoprotein (LDL), serum insulin, and hsCRP, which will be assessed using the fasting blood test results. We will calculate insulin resistance using the Homeostatic Assessment of Insulin Resistance (HOMA-IR) score (calculated as fasting glucose(mmol/L) × serum fasting Insulin(µU/L)/ 22.5; Lee et al., 2023). We will also look at non-conventional markers of vascular health as part of our secondary outcomes, such as central systolic and diastolic blood pressures, mean arterial pressure, augmentation index and pulse pressure. These are described separately in the Supplemental Material (see online Supplemental Material 2). Details on the primary and secondary outcome measures are provided in Table 4.

**Table 4.** Description of Measures.

Variable	Variable Measurement	Timepoint						
		Pregnancy^a^	1 Month	2 Months	3 Months	4 Months	5 Months	6 Months
Lactation Factors/Covariates								
Breastfeeding Intention^b^	Infant feeding intentions^1^		X					
Lactation Intensity	Monthly Infant Feeding Questionnaire^2^		X	X	X	X	X	
Breastfeeding Support	Monthly Questionnaire		X	X	X	X	X	
Demographics^c^								
Age		X						
Socioeconomic Index (SEIFA Score)^3,d^	All variables are self-reported/abstraction from medical records							X
Ethnicity		X						
Employment		X						X
Marital Status		X						X
Household Income		X						X
Cardiovascular Measurements								
Hemodynamics								
Peripheral and Central Blood Pressure (mmhg)	USCOM BP+ Device^4^							X
Augmentation Index (%)								X
Biochemical								
Total Cholesterol (mmol/L)	Blood test							X
Low Density Lipoprotein (mmol/L)								X
High Density Lipoprotein (mmol/L)								X
Triglycerides (mmol/L)								X
Serum Glucose (mmol/L)								X
Insulin (U/L)								X
High Sensitivity C-Reactive Protein (mmol/L)								X
Anthropometric								
Body Mass Index (kg/m^2^)		X						X
Waist Circumference (cm)								X
Medical History								
Medications	Self-report/abstraction	X						X
Familial History of Cardiometabolic Diseases (i.e., Type II Diabetes, Hypertension)	Self-report/abstraction	X						X
Previous History of Cardiometabolic Diseases	Self-report/abstraction	X						X
Diet and Exercise	Clinic Diet and Exercise Questionnaire^5^							X
Obstetric History								
Parity (N = Live Births)	All variables are self-reported/abstraction from medical records	X						
Gravidity (N = Pregnancies)		X						
Previous Pregnancy Complications		X						
Birthweight		X						
Gestational Age		X						
Plurality		X						
Gender		X						
Psychosocial Factors								
Antenatal Depression	Edinburgh Postnatal Depression Scale^6^	X						
Antenatal Anxiety	State and Trait Anxiety-Questionnaire^7^	X						
Antenatal Stress	Perceived Stress Questionnaire^8^	X						
	Antenatal Psychosocial Risk Questionnaire^9^	X						
Postpartum Depression	Patient Health Questionnaire-9^10^							X
Postpartum Anxiety	General Anxiety Disorder-7^11^							X
Postpartum Social Support	Medical Outcomes Survey (MOS)^12^							X

*Note.* Footnotes denote validation of assessment: 1. (Nommsen-Rivers & Dewey, 2009); 2. (Piper & Parks, 2001) 3. (ABS, 2023) 4.(Alberti et al., 2009) 5. (Aldridge et al., 2019) 6. (Cox et al., 1987) 7. (Austin et al., 2013) 8. (Cohen et al., 1983) 9. (Löwe et al., 2008) 10. (Kroenke et al., 2001) 11. (Auerbach, 1973) 12. (Sherbourne & Stewart, 1991).

a retrospective collection upon participant enrollment. ^b^collected during enrlment to study at 1 month postpartum. ^c^Demographics collected at 6-month time point to be reported descriptively. ^d^SEIFA Index of Relative Disadvantage (SEIFA-IRSD) score (where regions are scored as Quintiles 1–5, with Quintile 1 being the most disadvantaged).

To assess our exploratory aim, we will assess how lactation intensity is correlated with cardiometabolic and psychosocial variables during pregnancy, which will be abstracted from the participants’ medical records. We will assess cardiometabolic pregnancy variables such as use of anti-hypertensive and glucose-lowering medications, random glucose, early pregnancy BMI and blood pressure, and gestational weight gain. We will collect blood glucose readings (fasting, 1-hr and 2-hr glucose values) from the 75 g oral glucose tolerance test conducted at 24–28 weeks of gestation (or earlier if medically indicated due to GDM risk factors such as family history of T2DM). We will also assess the effect of antenatal mental health on lactation intensity using various screening questionnaires (highlighted in Table 4) administered as part of clinical care for depression, anxiety, antenatal psychosocial risk, and perceived stress. We will also assess different self-reported antenatal stressors such as financial strain and accommodation concerns, and drug and alcohol use during pregnancy. We will abstract obstetric and medical variables of the index pregnancy, including individual pregnancy complications, gravidity, parity, medical conditions during pregnancy, mode of delivery, age gestational age at delivery.

#### Planned data analysis

Data will be analyzed using SPSS (Version 29). Participant characteristics will be summarized using means and standard deviations, or median and interquartile range, for continuous variables, and frequencies and percentages for categorical variables. A point bi-serial correlation coefficient will be calculated to measure the strength of the association between the lactation intensity ratio and prevalence of metabolic syndrome at 6 months postpartum (binary = Yes or No). To assess our primary outcome (the association between lactation intensity and prevalence of metabolic syndrome) and our secondary outcomes (the relationship between lactation intensity, cardiovascular risk factors, and vascular dysfunction), we will compare cardiometabolic outcomes between different lactation intensity groups (i.e., high, medium and low intensities) through ANOVA. Logistic regression will be used to explore the effect of the lactation intensity score on metabolic syndrome prevalence. The model will be adjusted for age, socioeconomic status, BMI, smoking status, and other variables as listed in Figure 1. These variables have been selected ad-hoc as they are known to influence both metabolic syndrome prevalence and lactation intensity. The model will be based on both the continuous lactation intensity score and dichotomized ratio, where the score is assessed as high intensity (≥ 0.80) versus non-high intensity (≤ 0.79).

## Discussion

This study protocol highlights the first Australian observational study that will assess the relationship between lactation intensity and cardiovascular risk in people who experienced a major pregnancy complication at this early stage. Gunderson et al. (2012) reported on lactation intensity and postpartum impaired glucose tolerance in a cohort of American women with previous GDM, finding that those who exclusively breastfed at 6 to 9 weeks postpartum had lower serum glucose and insulin levels. Our study differs in that we are assessing lactation intensity across all major complications of pregnancy, and its effect on additional cardiovascular risk factors, including blood pressure and total cholesterol, which are known to be influenced by lactation (Burgess et al., 2021; Pathirana et al., 2023).

The rate of CVD in young people is higher than that of breast cancer, and strategies to reduce this risk are necessary for at-risk groups, such as those exposed to pregnancy complications. People who develop preeclampsia in pregnancy have a 2- to 4-fold increased risk of developing heart failure and stroke at an early age (R. Wu et al., 2020). Furthermore, people with previous GDM are at 7-fold increased risk of having T2DM (Juan et al., 2022). Therefore, this research will allow us to understand whether lactation is an effective method of CVD risk prevention that can be implemented into clinical guidelines for reducing premature heart disease and stroke.

A previous meta-analysis has shown that breastfeeding for any length of time following a pregnancy affected by GDM is associated with a reduction in blood glucose and risk of developing T2DM (Pathirana, Ali, et al., 2021). Furthermore, recently published data on an observational cohort of patients participating in a follow-up study in Australia showed that patients with at least one major complication of pregnancy who breastfed for at least 6 months had significantly lower systolic and diastolic blood pressure, lower serum insulin, and improved lipid profile compared to patients who did not breastfeed for at least 6 months (Pathirana et al., 2023). However, this study was limited by the data captured, as breastfeeding status was ascertained through the Child Health Record, or “blue book,” given to all South Australians when they give birth. Breastfeeding status is recorded dichotomously throughout infancy and early childhood and does not distinguish between mixed and exclusive feeding. Our study focuses on different intensities of lactation in the first 6 months postpartum and how this affects metabolic health in this similar population.

Metabolic syndrome has been shown to be prevalent in people who have had a complication of pregnancy, through identification of 40% of study participants with major pregnancy complications having MetS at 6 months postpartum, and a significant amount of participants with ≥ 1 risk factors, placing them on the trajectory of developing future coronary artery disease. Assessing lactation trends and lactation intensity in people with previous pregnancy complications is important to determine whether this improves their risk of developing MetS. In future, these results may aid in determining specific cardiovascular risk based on breastfeeding history, which can be considered when patients are provided with primary prevention advice. Additionally, it is known that low socioeconomic status and obesity is associated with reduced rates of exclusive breastfeeding (Carpay et al., 2021; Fan & Molinaro, 2021). Therefore, the prospective collection of detailed lactation data for this study may additionally aid local postnatal services in collectively trying to improve the length of lactation to benefit future cardiometabolic health.

## Limitations

There may be multiple lifestyle confounders impacting both breastfeeding and MetS. Furthermore, confounding lifestyle factors such as history of smoking and low educational status, which are prevalent in this cohort may influence lactation continuation. Smoking is a prominent risk factor for cardiometabolic diseases, and has been shown to affect breastmilk composition and an infant’s ability to latch (Napierala et al., 2016). Additionally, people who smoke are more likely to have shorter breastfeeding durations (Napierala et al., 2016). Low educational status influences understanding of health literacy and has been associated with low breastfeeding self-efficacy, as a person’s level of educational knowledge is correlated with their understanding of the health benefits of breastfeeding (Titaley et al., 2021). Therefore, the breastfeeding journey of each participant will not be linear due to the nature of these confounding factors.

In this study, we only identified lactation for a birthing parent’s own child. This may be a limitation as we are aware that pumping additional milk for donation is becoming more prevalent. However, as we know that there are barriers to breastfeeding in this cohort, we felt that adding a question about whether they provide breastmilk for other babies may be too confronting (particularly for mothers with small-for-gestational-age or growth restricted infants, who are likely recipients of donor milk). Future iterations of the survey could include a question regarding expressing breastmilk for other purposes.

We acknowledge that patients who have previously attended our clinic with previous pregnancy complications represent a group with a lower breastfeeding initiation rate than the national average. People with a history of pregnancy complications are likely to experience delayed lactogenesis and low milk supply (Horsley et al., 2022; J. L. Wu et al., 2021). This may be due to pre-existing cardiometabolic risk factors affecting lactation, and physical barriers due to the nature of a high-risk pregnancy (i.e., infant separation in Neonatal Intensive Care Unit; Horsley et al., 2022; Santoli et al., 2023; J. L. Wu et al., 2021). While the percentage of patients who initiate breastfeeding in our cohort is lower than the national average, we still believe that our cohort will represent patients on a spectrum of lactation intensity across 6 months. Assessing this real-world population will allow us to determine how these different levels of lactation affect postpartum metabolic health. We will also be able to assess the pregnancy variables that influence low lactation intensity (including individual pregnancy complications) through our exploratory analysis to identify people at high risk of poor breastfeeding outcomes, and target these factors to improve overall breastfeeding efficacy.

### Future Directions

The 6-month postpartum data will not distinguish participants who breastfed exclusively for 3 months from those who fed at 50% intensity for 6 months. To determine whether different feeding schedules that result in the same average intensity have an effect on outcomes, future analyses will focus on elucidating this further.

We will conduct a long-term assessment of this cohort until their 5-year postpartum follow-up appointment. This low socioeconomic cohort will be targeted for future consumer engagement to understand potential lifestyle and health-based barriers to lactation, which will link in accordingly with our secondary aim of assessing pregnancy factors influencing lactation. To assess how lactation intensity affects the anthropometric and hemodynamic health of the participants’ children, offspring assessments will also be conducted in the future, at 1 year, 3 years, and 5 years of age, to assess whether lactation could offset the negative obesogenic and inflammatory effects of exposure to an adverse intrauterine environment. The findings of this research will support a randomized controlled trial assessing improving breastfeeding self-efficacy and providing longer lactation support for reducing the development of metabolic syndrome.

## Supplemental Material

sj-docx-1-jhl-10.1177_08903344251396545 – Supplemental material for An Observational Study Protocol for Assessing Lactation Intensity and Reduction in the Prevalence of Metabolic Syndrome After a Maternal Complication of Pregnancy (LEMON Study) Supplemental material, sj-docx-1-jhl-10.1177_08903344251396545 for An Observational Study Protocol for Assessing Lactation Intensity and Reduction in the Prevalence of Metabolic Syndrome After a Maternal Complication of Pregnancy (LEMON Study) by Maleesa M. Pathirana, Prabha H. Andraweera, Emily Aldridge, Melanie R. Wittwer, Susan Sierp, Gustaaf Dekker and Margaret A. Arstall in Journal of Human Lactation
