# Supplementary material for: An Observational Study Protocol for Assessing Lactation Intensity and Reduction in the Prevalence of Metabolic Syndrome After a Maternal Complication of Pregnancy (LEMON Study)
Source: J Hum Lact. 2026 Feb 8;42(1):152–64. doi: 10.1177/08903344251396545 (PMC13013660; doi:10.1177/08903344251396545)
Supplement: sj-docx-1-jhl-10.1177_08903344251396545 – Supplemental material for An Observational Study Protocol for Assessing Lactation Intensity and Reduction in the Prevalence of Metabolic Syndrome After a Maternal Complication of Pregnancy (LEMON Study) [file sj-docx-1-jhl-10.1177_08903344251396545.docx]

**Appendix 1A: Infant Feeding Intentions Scale**

**Infant Feeding Intentions Scale**

Instructions: Below are some statements made about how mums will feed their babies. Please choose the answer that most closely matches your opinion, considering both your feeding plans and the likelihood that you will carry out those plans.

|  | Very much agree | Somewhat agree | Unsure | Somewhat disagree | Very much disagree |
| --- | --- | --- | --- | --- | --- |
| 1. I am planning to only formula feed (I will not breastfeed at all) 2. I am planning to at least give breastfeeding a try 3. When my baby is 1 month old, I will be breastfeeding without using any formula or other milk 4. When my baby is 3 months old, I will be breastfeeding without using any formula or other milk 5. When my baby is 3 months old, I will be breastfeeding without using any formula or other milk | 0 | 1 | 2 | 3 | 4 |
|  | 4 | 3 | 2 | 1 | 0 |
|  | 4 | 3 | 2 | 1 | 0 |
|  | 4 | 3 | 2 | 1 | 0 |
|  | 4 | 3 | 2 | 1 | 0 |

Researcher Score: ________________

*Adapted from Nommsen-Rivers and Dewey (2009) Development and validation of the infant feeding intentions scale*

**Appendix 1B: Infant Feeding Questionnaire**

**Infant Feeding Questionnaire:**

*This questionnaire is about your feeding over the past 7 days. Please answer as best as you can. You can use the notes section at the end of the questionnaire to make any comments if you need.*

***Today’s date:***

***Months postpartum:***

**Section A: General health**

1. In the past 7 days have you/are you suffering from the following? (please tick as many as applicable)
   1. Breast and nipple thrush
   2. Nipple discomfort (soreness)
   3. Poor latching
   4. Clogged milk ducts
   5. Mastitis
   6. Nipple vasospasm
   7. Low milk supply
   8. Oversupply
   9. Tongue tie
   10. Other (please list)
2. Are you currently on any medication for any breastfeeding conditions? (Provide medication name, dose and how often you have to take the medication (e.g. once a day, two times a day, three times and a week etc.).
3. Do you have any other medical problems and are taking other medication? (please list below)
4. If you are breastfeeding, what sort of support and resources are you accessing to help your feeding?
   1. No
   2. Yes – CAFHS
   3. Yes – Lactation Consultant
   4. Yes – Family advice and support
   5. Yes – Lactation cookies
   6. Yes – Other (please specify)

**SECTION B: Infant feeding**

1. How many times did you breastfeed your on baby on average over 24 hours? (This also excludes expressed feeding of breastmilk from a bottle) (*If it isn’t an exact number please round up to the closet integer)*
2. If you did breastfeed your baby over the last 7 days, how many hours are your feeds for each side on average for one feed? (*If a feed is only 15 minutes, write 0.25, 30 minutes write 0.5, 45 minutes 0.75)*
   1. Side 1:
   2. Side 2:
3. How many times did you formula feed your baby on average over 24 hours? (If this isn’t an exact number please round up to the closest integer)
4. How many times did you feed your baby solid foods (e.g. pouches, puree food, soft foods) (If this isnt exact please round up to the closest integer)

**Do you have any other questions/comments you would like to pass on to the research team? 😊**

**…………………………………………………………………………………………………………………………………………………….**

**…………………………………………………………………………………………………………………………………………………….**

**…………………………………………………………………………………………………………………………………………………….**

**…………………………………………………………………………………………………………………………………………………….**

**…………………………………………………………………………………………………………………………………………………….**

**…………………………………………………………………………………………………………………………………………………….**

**Appendix 2: Description of study measures**

**Description of measures:**

1. *Patient Health Questionnaire-9*

This is a self-administered questionnaire that screens for major depressive disorder. The 10-item questionnaire uses Likert scale ratings for screening depression and severity of depressive symptoms

1. *General Anxiety Disorder Questionnaire-7*

This is a self-administered questionnaire screening for generalised anxiety disorder. This is a 7-item questionnaire uses Likert scale ratings for screening anxiety and severity of anxiety symptoms.

1. *Medical Outcomes Survey*

This is a validated self-report frequency scale that sub-groups support into four categories: emotional/information support, tangible support, affectionate support and positive social interaction.

1. *Diet and Exercise Questionnaire*

This is an administered questionnaire that asks about weekly and/or daily frequencies of dairy, protein, vegetables, fruit, discretionary food, added sugars, wholemeal and other carbohydrate, water intake and hours of exercise per day.

1. *USCOM BP+*

This device has been validated against gold-standard aneroid blood pressure measurements and is maintained by the COFFEE Clinic. Patients after 20 minutes of resting have their cuff size measured based on arm circumference and their blood pressure is taken. Only readings of “Excellent” or “Good” quality as reported on the USCOM BP+ are included.

1. *Antenatal (psychosocial) Risk Questionnaire (ANRQ)(Austin, Colton, Priest, Reilly, & Hadzi-Pavlovic, 2013)*

Risk of perinatal mental health morbidity. A score Women were considered at high risk with a score >22 or if they answered yes to any of the following questions: 2A (“have you ever had 2 weeks or more where you felt particularly worried, miserable or depressed?), 2B (do you have any other history of mental health problems?) 8 (were you emotionally abused growing up?), 9 (have you ever been sexually or physically abused?)

1. *Edinburgh Postnatal Depression Score (EPDS)(Cox, Holden, & Sagovsky, 1987)*
   Assesses likelihood of depression. “low risk” of depression was scored 0-9, “moderate risk” of depression in the following year score 10-12, and “likely depressed” score 13-30.
2. *State and Trait Anxiety score-6 (STAI-6) (Auerbach, 1973)*
   Assessment of anxiety. A score below 30 is defined as “low to no anxiety”, 31-49 “normal level of anxiety” and a score of 45-80 is defined as a participant having an “elevated state of anxiety*”*
3. *Perceived Stress Questionnaire (PSS) (Cohen, Kamarck, & Mermelstein, 1983)*

A score between 0-13 was considered “low” stress, 14-26 “moderate” stress and 27-40 “high” perceived stress.
